# Irreducible Anterior Shoulder Dislocation with Interposition of the Long Head of the Biceps and Greater Tuberosity Fracture: A Case Report and Review of the Literature

**DOI:** 10.2174/1874325001711010327

**Published:** 2017-04-27

**Authors:** Konstantinos Pantazis, Andreas Panagopoulos, Irini Tatani, Basilis Daskalopoulos, Ilias Iliopoulos, Minos Tyllianakis

**Affiliations:** Department of Shoulder & Elbow Surgery, Patras University Hospital, Patras, Greece

**Keywords:** Irreducible, Anterior, Shoulder dislocation, Long head of biceps, Greater tuberosity fracture, Open reduction

## Abstract

**Background:**

Failure of closed manipulative reduction of an acute anterior shoulder dislocation is seldom reported in the literature and is usually due to structural blocks such as soft tissue entrapment (biceps, subscapularis, labrum), bony fragments (glenoid, greater tuberosity) and severe head impaction (Hill-Sachs lesion).

**Case report:**

We present a case of an irreducible anterior shoulder dislocation in a 57-year-old male patient after a road-traffic accident. He had severe impaction of the head underneath glenoid rim and associated fracture of the greater tuberosity. Closed reduction performed in the emergency room under sedation and later at the theatre under general anaesthesia was unsuccessful. Open reduction using the dectopectoral approach revealed that the reason for obstruction was the posterolateral entrapment of the biceps tendon between the humeral head and the tuberosity fragment. Reduction was achieved after subscapularis tenotomy and opening of the joint; the tuberosity fragment was fixed with transosseous sutures and the long head of the biceps tendon was tenodesized. The patient had an uneventful postoperative recovery and at his last follow up, 12 months postoperatively, he had a stable joint, full range of motion and a Constant score of 90.

**Conclusion:**

A comprehensive literature review revealed 22 similar reports affecting a total of 30 patients. Interposition of the LHBT alone or in combination with greater tuberosity fracture was the most common obstacle to reduction, followed by subscapularis tendon interposition and other less common reasons. Early surgical intervention with open reduction and confrontation of associated injuries is mandatory for a successful outcome.

## INTRODUCTION

The shoulder is potentially one of the most unstable joints of the body, with very little bony stability or containment, adhering a fine balance between the mobility to perform athletic activities and stability required to power and stabilise the arm. Both static (glenoid labrum, glenoid concavity, glenohumeral ligaments, vacuum effect) and dynamic (proprioception, periarticular musculature) stabilizers work in synchrony to maintain stability in performing the extreme activities required by the shoulder in sports and heavy manual work [1]. Despite that, owing to the wide range of shoulder’s motion, it is the most susceptible joint to dislocation in human body. Dislocations can occur anteriorly, posteriorly or inferiorly, however the most frequent dislocations are anterior, accounting for approximately 96% [2]. The vast majority of them are treated with closed reduction under light sedation or general anesthesia.

The clinical presentation of anterior shoulder dislocation is usually obvious. Patients support their affected shoulder in a slightly abduction and external rotation. The clinician must confirm distal pulses and rule out neurological injuries, although neurovascular injuries are not contraindication for closed reduction [3]. Radiographic documentation of the dislocation as well as possible associated osseous injuries should be performed before attempting reduction. Numerous methods of reduction have been described in the literature, using traction, leverage or scapular manipulation. Success rates of 70% to 90% have been reported, regardless of the technique used [4].

Irreducible anterior shoulder dislocation is a rare entity requiring open reduction. Structural blocks to reduction include soft tissue entrapment (biceps, subscapularis), bony fragments (glenoid, greater tuberosity) and severe head impaction (Hill-Sachs). Presented here is a case of an acute irreducible anterior shoulder dislocation due to interposed long biceps tendon and greater tuberosity fracture. A comprehensive review of the recent literature is provided as well.

## CASE REPORT

**Fig. (1):**
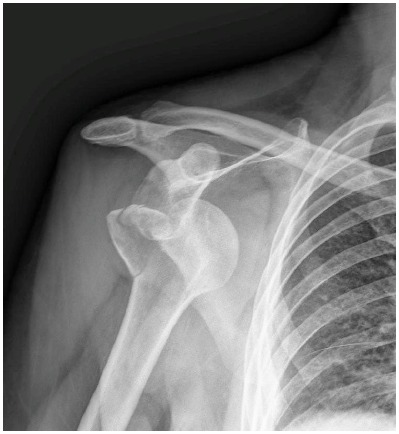
Anteroposterior view of the right shoulder showing anterior dislocation with impaction of the humeral head (Hill-Sachs lesion) and associated comminuted greater tuberosity fracture.

**Fig. (2):**
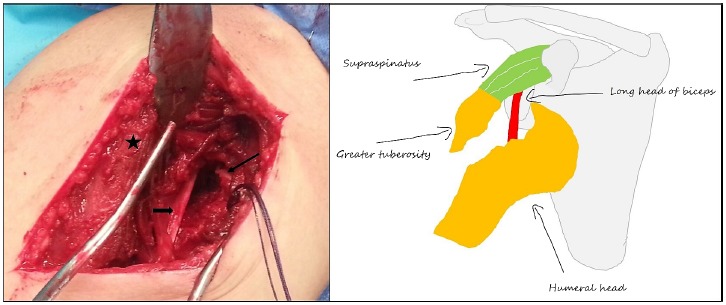
Intraoperative view of the dislocation through the dectopectoral approach. The thin arrow shows the impaction of the humeral head underneath glenoid rim, the small arrow the interposed long head of the biceps tendon and the star the greater tuberosity fragment. The drawing at the right illustrates the intraoperative findings.

**Fig. (3):**
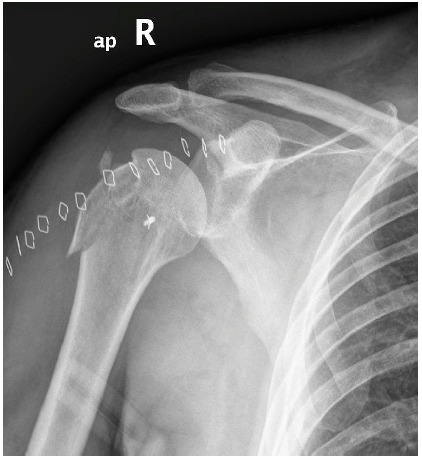
Postoperative anteroposterior view of the right shoulder showing reduction of the dislocation and fixation of the greater tuberosity with transosseous sutures. The bone anchor indicates the site of biceps tendon tenodesis.

**Fig. (4):**
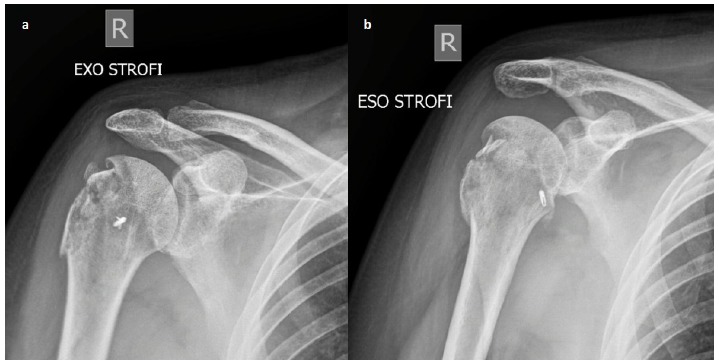
Follow up anteroposterior x-ray of the right shoulder in external (a) internal (b) and rotation showing a stable joint and good healing of the greater tuberosity.

A 57-year-old male patient was transferred to the emergency department after a road traffic accident. He had a fall from his bike and his right arm was crashed by the following car. On clinical examination, he had pain and obvious deformity in the shoulder while he was unable to move his arm in any direction, especially in external rotation. He had numbness in the regimental badge region of the axillary nerve and normal radial pulse at the wrist. Radiological examination showed an anterior shoulder dislocation with impaction of the humeral head underneath glenoid and associated fracture of the greater tuberosity (Fig. **1**). He had no other skeletal or visceral injuries. After conscious sedation, an effort for closed reduction (3 attempts) was performed in the emergency room but was unsuccessful. For reasons unrelated to the patient or the disease a CT-scan of the shoulder was not possible to be performed at that time. The patient was transferred immediately to the operative theatre for closed manipulation under general anaesthesia in order to reduce the dislocation and avoid further neurological compromise; a CT-scan and/or MRI has been scheduled for the next morning. Despite prompt sedation and muscle relaxation the shoulder was still unable to interpose. Open reduction was accomplished thereafter using the deltopectoral approach; the biceps tendon was found entrapped posterolateral between the humeral head and the tuberosity fragment preventing once again reduction of the glenohumeral joint. (Fig. **2**). Tenotomy and tenodesis of the frayed biceps at the level of pectoralis major was performed using a bone anchor; the transverse ligament at the bicipital groove had been torned. Next, the subscapularis was incised, 1 cm medial to its insertion at the lesser tubercle, and retracted medially after separated from the capsule. The latter was incised and the labrum was clearly visualized and was found to be intact inferomedially. Using longitudinal traction and finger manipulation in an anterior direction the humeral head was finally reduced revealing a large triangular Hill-Sachs lesion at its posterior part. The greater tuberosity was fixed back to its bed using heavy transosseous sutures (Fig. **3**). The capsule and subscapularis tendon were repaired anatomically with sutures and the shoulder joint was found stable through a full range of motion of internal rotation and external rotation with the arm in adduction and at 90° of abduction. The patient had an uneventful postoperative recovery without neurovascular compromise. Pendulum exercises initiated from the second postoperative day followed by passive assisted forward flexion and limitation of active internal rotation for 4 weeks. At the last follow up, one year postoperatively he had a stable joint, full range of motion and a Constant score of 90 (Fig. **4**).

## DISCUSSION

Most acute anterior shoulder dislocations are easily reduced by closed means. There are a few cases of irreducible dislocations reported in the literature [5-26], usually affecting young men at their fourth decade of life. Mechanical obstructers preventing reduction have been attributed to large Hill-Sachs lesions and interposition of the subscapularis or the long-head of the biceps. Other less common causes include free fracture fragments, torn labrum in the glenoid rim and entrapment of surrounding nerves. In our case, there was a combination of factors preventing closed reduction: the severely impacted head at the inferior margin of the glenoid with the associated Hill- Sachs lesion, the large greater tuberosity fragment and the interposition of the long head of the biceps tendon (LHBT) between the tuberosity fragment and the posterolateral aspect of the humeral head.

A comprehensive review of the literature revealed 22 reports of irreducible anterior shoulder dislocation affecting a total of 30 patients [5-26] (Table **1**). Nakhaei Amroodi M [15] reported on 7 patients; 6 had greater tuberosity fracture and LHBT interposition and one a “shield” greater and lesser tuberosity fracture interposed by subscapularis and infraspinatus tendons. Seradge H & Orme G [7] reported on 3 cases with 3 different mechanisms of obstruction; the rest 20 reports concerned single cases only. Interposition of the LHBT alone or in combination with greater tuberosity fracture was the most common obstacle to reduction [5, 7, 9, 11-16], followed by subscapularis tendon interposition either tightened around the humeral head or as soft tissue hindrance inside glenoid [17, 19-24]. Other reasons for an unsuccessful closed reduction were the interference of bony fragments [18, 25] or the labrum [7] inside glenoid, the presence of a “shield” fracture of both tuberosities [15], interposition of the musculocutaneus nerve [26] and two cases of anterosuperior dislocation with torn rotator cuff and the humeral head lying underneath deltoid muscle [8, 10].

Posterolateral subluxation of the biceps tendon as part of an irreducible anterior shoulder dislocation was the most common mechanical blockage and was usually associated with high-energy trauma to the glenohumeral joint and associated displaced fracture of the greater tuberosity. In the absence of greater tuberosity fracture, in order posterior subluxation to occur, other stabilizing structures such as supraspinatus and infraspinatus tendons and the transverse ligament of the bicipital groove must be torn. The high energy acting at the time of the dislocation drives the LHBT slipped posteriorly and inside the joint space hanging around the humeral head. In addition to this pathology, a large Hill-Sachs defect locks the head at the inferior glenoid rim further hindering closed reduction. In such cases, an open reduction with tenotomy of the lacerated LHBT in order to aid reduction and tenodesis at the sight of pectoralis major must be considered. Bahrs *et al.* [27] associated avulsion fractures of the GT during anterior shoulder dislocation at 10-30% of cases. The overall incidence of displaced GT fracture as part of an anterior dislocation in a series of 544 cases of proximal humeral fractures that underwent operative treatment in our clinic between 1993 and 2002 was 6.6% [28]. The suggested mechanism is impingement of the GT against the acromion and the glenoid rim presented as a complete form of Hill-Sachs lesion. In our center, we manage displaced GT fractures using heavy transosseous sutures as they provide adequate stabilization with minimal soft-tissue damage and low risk for further fragment comminution [29].

The second most common cause in irreducible anterior shoulder dislocations is subscapularis tear or avulsion. In most of the cases, the tendon is strained around the anterior aspect of the joint keeping the humeral head impacted underneath the anterior rim of the glenoid with a large Hill-Sachs lesion. Otherwise, the torn or avulsed tendon with its lesser tuberosity fragment is apposed between glenoid and humeral head opposing closed reduction. Concurrent rotator cuff tear is another important contributing factor, presented in 30% of shoulder dislocations in patients older than forty years old [30]. Additionally, the inferior glenohumeral ligament (IGHL) may be also torn; its superior fibers together with the subscapularis tendon are the main soft tissue stabilizers at 0°-30°of abduction preventing anterior dislocation of the humeral head [31].

Conventional radiographic evaluation could successfully prognoses the easiness of closed reduction. Suspicion for posterior or lateral LHBT dislocation can be raised when the displaced humeral head is medial to the coracoid process or when a large greater tuberosity fracture coexists with more than 1 cm displacement from the humeral head [32]. Plain anteroposterior and true lateral scapular views are usually adequate; Stryker notch view, although difficult to obtain, can identify small Hill-Sachs lesions. CT with 3D reconstruction provides an accurate demonstration of the injury facilitating the reduction technique using the appropriate maneuvers [15, 33]. In our case, CT-scan was temporarily unavailable at the time of admission but considering the course of the disease and the need for open reduction we don’t believe that this exam would offer any additional benefit to the final outcome. MRI offer significant information but is commonly ordered after reduction in order to assess stability. MRI is indicated for soft tissue lesions like RC tears, whereas MRI arthrography is superior when assessing posterolateral dislocation of LBHT.

The incidence of nerve lesions following anterior fracture dislocation of the shoulder has been estimated up to 55%, with the axillary nerve most commonly involved usually in the form of neurapraxia [34, 35]. Our patient showed numbness at the regimental badge region of the axillary nerve, completely resolved after reduction. Other nerves reported to be involved are the suprascapular, the radial, the musculocutaneous as well as the brachial plexus. Traction is the commonest mechanism of injury, as the nerve is tightened over the dislocated humeral head. Lesions may occur also during reduction maneuvers especially when excessive traction and external rotation are applied at the same time. In cases of irreducible shoulder dislocations, manipulative maneuvers must be limited and performed with great caution having in mind the possible interposition of nerves. Major vessels are not damaged in anterior shoulder dislocations. The axillary artery may be compromised in neglected fracture dislocations in patients older than 50 years old [36].

## CONCLUSION

Acute irreducible anterior shoulder dislocation is not very common, even though must be suspected when associated with fracture of the greater tuberosity. Structural blocks to reduction may include soft tissue entrapment (biceps, subscapularis, labrum, nerves), bony fragments (glenoid, greater tuberosity) and severe head impaction (Hill-Sachs). Repeated forceful manipulation can increase the risk of fracture or neurovascular injury and should be avoided. Two plain x-rays must always be acquired before reduction, whereas in cases of uncommon bony fragments and impacted humeral head a CT or MRI scan should be ordered. Early surgical intervention with open reduction and confrontation of associated injuries is mandatory for a successful outcome.

## Figures and Tables

**Table 1:** Comprehensive literature review of irreducible anterior shoulder dislocations.

Author	Year	Cause of obstruction	Cases
Bridle S.H. [23]	1990	Interposition of subscapularis	1
Connolly S. [24]	2007		1
Richard C.S. [19]	2007		1
Inao S. [12]	1990	Interposition of LHBT	1
Mullaney P.J. [13]	2007		1
**Seradge H. [7]**	1982		1
Strobel K. [14]	2002		1
Day M.S. [6]	2010		1
Lam S. [20]	1966	Hill - Sachs lesion and subscapularis bowstringing	1
Kuhnen W. [21]	1979		1
Guha A.R. [22]	2004		1
Aiyenuro O.D. [17]	2007		1
**Seradge H. [7]**	1982	Greater tuberosity fracture and interposition of LHBT	1
*Nakhaei Amroodi M. [15]*	2015		6/7
Janecki C.J. [9]	1979		1
Davies M.B. [16]	2000		1
Ilahi O.A. [11]	1998		1
Henderson R.S. [5]	1952		1
**Seragde H. [7]**	1982	Interposition of the labrum inside glenoid	1
*Nakhaei Amroodi M. [15]*	2015	“Shield” fracture of GT and LT with interposition of subscapularis and infraspinatus	1/7
Mihata T. [25]	2000	Bony fragment inside glenoid	1
Oni O.A. [18]	1983		1
Gudena R.[26]	2011	Muscolocutaneus nerve	1
Wyatt A.R. [10]	2015	Rotator cuff tear and anterosuperior dislocation	1
Tietjen R. [8]	1982		1

LHBT: Long Head of the Biceps Tendon, GT: Greater Tuberosity, LT: Lesser Tuberosity

## LIST OF ABBREVIATIONS

GT: = Greater Tuberosity
IGHL: = Inferior GlenoHumeral Ligament
LHBT: = Long Head of the Biceps Tendon
